# Computation of Nevanlinna characteristic functions derived from generating functions of some special numbers

**DOI:** 10.1186/s13660-018-1722-y

**Published:** 2018-06-07

**Authors:** Serkan Araci, Mehmet Acikgoz

**Affiliations:** 1 Department of Economics, Faculty of Economics, Administrative and Social Sciences, Hasan Kalyoncu University, Gaziantep, Turkey; 2 Department of Mathematics, Faculty of Arts and Science, University of Gaziantep, Gaziantep, Turkey

**Keywords:** 11B68, 30D30, 30D35, Meromorphic function, Poisson–Jensen formula, Nevanlinna characteristic function, Normal function, Bernoulli numbers, Associated Euler numbers, Generating function

## Abstract

In the present paper, firstly we find a number of poles of generating functions of Bernoulli numbers and associated Euler numbers, denoted by $n ( a,\mathbf{B} ) $ and $n ( a,E ) $, respectively. Secondly, we derive the mean value of a positive logarithm of generating functions of Bernoulli numbers and associated Euler numbers shown as $m ( 2\pi,\mathbf{B} ) $ and $m ( \pi,E ) $, respectively. From these properties, we find Nevanlinna characteristic functions which we stated in the paper. Finally, as an application, we show that the generating function of Bernoulli numbers is a *normal function*.

## Introduction and preliminaries

In the mathematical field of complex analysis, Nevanlinna theory deals with the theory of meromorphic functions. It was constructed in 1925 by Finnish mathematician Rolf Herman Nevanlinna (October 22, 1895–May 28, 1980), who made significant contributions to complex analysis. Because of devising of R. Nevanlinna, Hermann Weyl has called it “one of the few great mathematical events of (the twentieth) century” [1]. In fact, Nevanlinna theory plays an important role in transcendental meromorphic functions, analytic theory of differential and functional equations, holomorphic dynamics, minimal surfaces, and complex hyperbolic geometry, which deals with generalizations of Picard’s theorem to higher dimensions, *cf.* [1–8] and the references cited therein.

Nevanlinna theory defines the asymptotic distribution of solutions of the equation

$$ f(z)=a. $$

In this theory, a fundamental tool is the Nevanlinna characteristic given by

$$ T ( r,f ) =T \biggl( r,\frac{1}{f} \biggr) +\log \bigl\vert f ( 0 ) \bigr\vert , $$

which measures the rate of growth of a meromorphic function, *cf.* [1–7].

We now begin with the properties of Nevanlinna theory.

### Theorem 1

*Let* $f ( z ) $ *be a meromorphic function in* $\vert z \vert \leq R$ ($0< R<\infty$), *and let* $a_{i}$ ($i=1,2,\ldots,M$), $b_{j}$ ($j=1,2,\ldots ,N$) *be the zeros and poles of* $f ( z ) $ *in* $\vert z \vert < R$, *respectively*. *If* $z=re^{i\theta}$ ($0< r< R$) *is a point in* $\vert z \vert < R$, *distinct from* $a_{i}$ *and* $b_{j}$, *then*

$$\begin{aligned} \log \bigl\vert f ( z ) \bigr\vert =&\frac{1}{2\pi}\int_{0}^{2\pi}\log \bigl\vert f \bigl( Re^{i\phi} \bigr) \bigr\vert \frac{R^{2}-r^{2}}{R^{2}-2Rr\cos ( \theta-\phi ) +r^{2}}\, d\phi \\ &{}+\sum_{i=1}^{M}\log \biggl\vert \frac{R ( z-a_{i} ) }{R^{2}-\overline{a}_{i}z} \biggr\vert -\sum_{j=1}^{N} \log \biggl\vert \frac {R ( z-b_{j} ) }{R^{2}-\overline{b}_{j}z} \biggr\vert , \end{aligned}$$

*which is called Poisson–Jensen formula*, *see* [3].

### Definition 1

Let $n ( R,f ) $ denote a number of poles in $\vert z \vert \leq R$ so that $n ( R,\frac {1}{f} ) $ denotes a number of zeros in $\vert z \vert \leq R$. These values are known as

$$\begin{aligned}& N ( R,\infty ) := N ( R,f ) =\sum_{j=1}^{N} \log\frac{R}{ \vert b_{j} \vert }= \int_{0}^{R}n ( t,f ) \frac {dt}{t}, \\& N ( R,0 ) := N \biggl( R,\frac{1}{f} \biggr) =\sum _{i=1}^{M}\log \frac{R}{ \vert a_{i} \vert }= \int_{0}^{R}n \biggl( t,\frac {1}{f} \biggr) \frac{dt}{t}\quad \text{(see [3])}. \end{aligned}$$

From Definition 1, one may write

1.1 $$ N ( R,f ) = \int_{0}^{R}n ( t,f ) \frac{dt}{t}, $$

which is called *Nevanlinna’s counting function*.

### Proposition 1

*If* $f ( 0 ) =\infty$,

$$ N ( R,f ) = \int_{0}^{R} \bigl( n ( t,f ) -n ( 0,f ) \bigr) \frac{dt}{t}+n ( 0,f ) \log R\quad (\textit{see }\mbox{[3]}). $$

### Proposition 2

*If* $f ( 0 ) =0$, *under the same conditions of Theorem *1, *then*

$$ f ( z ) =\sum_{k=m}^{\infty}C_{k}z^{k} \quad ( C_{m}\neq0\textit{ with }m\in \mathbb{Z} ) . $$

*In fact*, $m>0$ *if the origin is a zero of order* *m*, *and* $m<0$ *if the origin is a pole of order* *m*. *Then the following holds true*:

$$ \log \vert C_{m} \vert =\frac{1}{2\pi} \int_{0}^{2\pi }\log \bigl\vert f \bigl( re^{i\theta} \bigr) \bigr\vert \,d\theta -\sum _{i=1}^{M}\log\frac{ \vert a_{i} \vert }{R}+\sum _{j=1}^{N}\log\frac{ \vert b_{j} \vert }{R}-m\log R \quad(\textit{see }\mbox{[3]}). $$

### Definition 2

Let *x* be a positive real number. The positive logarithm log^+^ is defined by (see [3])

$$ \log^{+}x=\max \{ \log x,0 \} = \textstyle\begin{cases} \log x, & \text{if }x>1, \\ 0, & \text{if }x\leq1. \end{cases} $$

Notice that the positive logarithm defined above is a continuous function of nonnegative on $( 0,\infty ) $.

### Corollary 1

*From Definition *1, *one has*

$$ \log x=\log^{+}x-\log^{+}\frac{1}{x}\quad ( \textit{see }\mbox{[3]}). $$

Then we can easily derive the following integral equation from Corollary 1:

1.2 $$ \int_{0}^{2\pi}\log \bigl\vert f \bigl( Re^{i\theta} \bigr) \bigr\vert \,d\theta= \int_{0}^{2\pi}\log^{+} \bigl\vert f \bigl( Re^{i\theta } \bigr) \bigr\vert \,d\theta- \int_{0}^{2\pi}\log^{+}\frac{1}{ \vert f ( Re^{i\theta} ) \vert }\,d \theta. $$

### Theorem 2

*If* $f ( 0 ) \neq0,\infty$, *one has*

$$\begin{aligned} \log \bigl\vert f ( 0 ) \bigr\vert =&\frac{1}{2\pi} \int _{0}^{2\pi }\log^{+} \bigl\vert f \bigl( re^{i\theta} \bigr) \bigr\vert \,d\theta-\frac{1}{2\pi} \int_{0}^{2\pi}\log^{+}\frac{1}{ \vert f ( re^{i\theta } ) \vert }\,d \theta \\ &{}+N ( r,f ) -N \biggl( r,\frac {1}{f} \biggr)\quad (\textit{see }\mbox{[3]}). \end{aligned}$$

### Definition 3

The Nevanlinna characteristic function of $f ( z ) $, denoted by $T ( r,f ) $, is given by

$$ T ( r,f ) =N ( r,f ) +m ( r,f ), $$

where $m ( r,f ) $ is mean value of the function $\log ^{+} \vert f ( re^{i\theta} ) \vert $ on $[ 0,2\pi ] $ (see [3]).

### Theorem 3

*Jensen–Nevanlinna formula is known by*

$$ T ( r,f ) =T \biggl( r,\frac{1}{f} \biggr) +\log \bigl\vert f ( 0 ) \bigr\vert \quad (\textit{see }\mbox{[3]}). $$

The works on special numbers and polynomials have a very long history. In fact, special numbers and polynomials play a significantly important role in the progress of several fields of mathematics, physics, and engineering. They have many algebraic operations. That is, because of their finite evaluation schemes and closure under addition, multiplication, differentiation, integration, and composition, they are richly utilized in computational models of scientific and engineering problems. For more information related to special numbers and polynomials, see [9–11] and the references cited therein.

By this motivation, we find a number of poles of generating functions of Bernoulli numbers and associated Euler numbers, denoted by $n ( a,\mathbf {B} ) $ and $n ( a,E ) $, respectively. After that, we derive the mean value of a positive logarithm of generating functions of Bernoulli numbers and associated Euler numbers shown as $m ( 2\pi,\mathbf{B} ) $ and $m ( \pi ,E ) $, respectively. From these properties, we find Nevanlinna characteristic functions which we stated in the following parts. In the final part of this paper, as an application, we show that the generating function of Bernoulli numbers is a *normal function*.

## Nevanlinna characteristic function of generating function of Bernoulli numbers

Let $B_{n} ( x ) $ be Bernoulli polynomials defined by means of the following generating function:

$$ B(x,z)=\frac{z}{e^{z}-1}e^{xz}=\sum_{n=0}^{\infty}B_{n} ( x ) \frac{z^{n}}{n!}\quad \bigl( \vert z \vert < 2\pi \bigr) . $$

In the case when $x=0$, we have $B_{n} ( 0 ) :=B_{n}$ that stands for Bernoulli numbers expressed by the following generating function (*cf.* [9–11]):

$$ B(z)=\frac{z}{e^{z}-1}=\sum_{n=0}^{\infty}B_{n} \frac{z^{n}}{n!}. $$

Here we first consider the generating function of Bernoulli numbers $B(z)$. One of the zeros of $B(z)$ is $z=0$. From here, we see that

$$ \lim_{z\rightarrow0}B ( z ) =1. $$

It means $B ( z ) $ has a removable singular point at $z=0$. Then we have the following corollary.

### Corollary 2

*The function* $B ( z ) $ *is not a meromorphic function over complex plane including* $z=0$.

We now modify the generating function of Bernoulli numbers as follows:

2.1 $$ \mathbf{B} ( z ) =\frac{1}{z}B(z)=\frac{1}{e^{z}-1}= \frac {1}{z}+\sum_{n=0}^{\infty} \frac{B_{n+1}}{n+1}\frac{z^{n}}{n!}. $$

### Corollary 3

*The function* $\mathbf{B} ( z ) $ *is a meromorphic function at everywhere*.

Let us now consider $\mathbf{B} ( z ) $ over the following disk:

$$ D= \bigl\{ z\in \mathbb{C} \mid \vert z \vert \leq a \bigr\} . $$

The function $\mathbf{B} ( z ) $ has no zeros. However, it has poles as follows:

$$ e^{z}-1=0\quad \Rightarrow\quad e^{z}=e^{2k\pi i} \quad \Rightarrow\quad z=2k\pi i \quad ( k\in \mathbb{Z} ) . $$

A number of poles over disk *D* are as follows:

- If $a=\pi$, the pole is 0: that is, $n ( a,\mathbf {B} ) =1$ where $\mathbf{B}:=\mathbf{B} ( z ) $.
- If $a=2\pi$, the poles are $-2\pi i$, 0, $2\pi i$: that is, $n ( a,\mathbf{B} ) =3$.

Then we have the following corollary.

### Corollary 4

*A number of poles of the function* $\mathbf{B} ( z ) $ *over disk* $D= \{ z\in \mathbb{C} \mid \vert z \vert \leq a \} $

$$ n ( a,\mathbf{B} ) =1+2 \biggl[ \frac{a}{2\pi} \biggr], $$

*where the notation* $[ x ] $ *denotes the largest integer less than or equal to* *x*.

Now we give the following theorem.

### Theorem 4

*The function* $\mathbf{B} ( z ) $ *holds for* $a=2\pi$ *over disk* *D*

$$ N ( 2\pi,\mathbf{B} ) =\log ( 2\pi ) . $$

### Proof

Since $\mathbf{B} ( 0 ) =\infty$, it follows from Proposition 1 that

$$ N ( 2\pi,\mathbf{B} ) = \int_{0}^{2\pi} \bigl( n ( t,\mathbf{B} ) -n ( 0,\mathbf{B} ) \bigr) \frac{dt}{t}+n ( 0,\mathbf{B} ) \log ( 2 \pi ) . $$

When $n ( 0,\mathbf{B} ) =1$, we write

$$ N ( 2\pi,\mathbf{B} ) =2 \int_{0}^{2\pi} \biggl[ \frac {t}{2\pi} \biggr] \frac{dt}{t}+\log ( 2\pi ) . $$

When $[ \frac{t}{2\pi} ] =0$ on $[ 0,2\pi ) $, we deduce

$$ N ( 2\pi,\mathbf{B} ) =\log ( 2\pi ), $$

which completes the proof. □

### Theorem 5

*The mean value of the function* $\mathbf{B} ( z ) $ *on* $[ 0,2\pi ) $ *is that*

$$ m ( 2\pi,\mathbf{B} ) =O ( 1 ), $$

*where* $O ( \cdot ) $ *means big* *O* *notation*; *for information about this notation*, *see* [3].

### Proof

Setting $a=2\pi$ gives $z=2\pi e^{i\theta}=2\pi ( \cos\theta +i\sin \theta ) $. By the triangle inequality $\vert \vert z_{1} \vert - \vert z_{2} \vert \vert \leq \vert z_{1}-z_{2} \vert $, we have

$$\begin{aligned} \bigl\vert e^{z}-1 \bigr\vert =& \bigl\vert e^{2\pi ( \cos \theta +i\sin\theta ) }-1 \bigr\vert \\ \geq& \bigl\vert \bigl\vert e^{2\pi\cos\theta}e^{i\sin\theta } \bigr\vert - \vert 1 \vert \bigr\vert \\ =& \bigl\vert \bigl\vert e^{2\pi\cos\theta} \bigr\vert \bigl\vert e^{i\sin\theta} \bigr\vert -1 \bigr\vert \\ =& \bigl\vert e^{2\pi\cos\theta}-1 \bigr\vert . \end{aligned}$$

From here, we obtain the following useful inequality:

$$ \log^{+}\frac{1}{ \vert e^{z}-1 \vert }\leq\log^{+}\frac {1}{ \vert e^{2\pi\cos\theta}-1 \vert }. $$

Then we derive the mean value of the function $\mathbf{B} ( z ) $ on $[ 0,2\pi ) $

$$\begin{aligned} m ( 2\pi,\mathbf{B} ) =&\frac{1}{2\pi} \int_{0}^{2\pi }\log^{+}\frac{1}{ \vert e^{z}-1 \vert }\,d\theta \\ \leq&\frac{1}{2\pi} \int_{0}^{2\pi}\log^{+}\frac{1}{ \vert e^{2\pi \cos\theta}-1 \vert }\,d \theta. \end{aligned}$$

Since

$$ e^{2\pi\cos\theta}-1=2\pi\cos\theta+\frac{ ( 2\pi\cos \theta ) ^{2}}{2!}+\cdots $$

with $\vert \cos\theta \vert \geq0$, we find

$$ \bigl\vert e^{2\pi\cos\theta}-1 \bigr\vert \geq2\pi \vert \cos \theta \vert \geq \vert \cos\theta \vert . $$

Then we have

$$\begin{aligned} m ( 2\pi,\mathbf{B} ) \leq&\frac{1}{2\pi} \int _{0}^{2\pi}\log ^{+}\frac{1}{ \vert \cos\theta \vert } \,d\theta \\ =&\frac{1}{2\pi} \int_{0}^{2\pi}\log\frac{1}{ \vert \cos \theta \vert }\,d\theta. \end{aligned}$$

Here, when the integral $\frac{1}{2\pi}\int_{0}^{2\pi}\log\frac {1}{ \vert \cos\theta \vert }\,d\theta$ is continuous, we get

$$ m ( 2\pi,\mathbf{B} ) =O ( 1 ), $$

which is the desired result. □

### Theorem 6

*The Nevanlinna characteristic function of the function* $\mathbf {B} ( z ) $ *is that*

$$ T ( 2\pi,\mathbf{B} ) =\log ( 2\pi ) +O ( 1 ) . $$

### Proof

Since it follows from Definition 3, Theorem 4, and Theorem 5, we omit the proof. □

## Nevanlinna characteristic function of generating function of associated Euler numbers

The Euler polynomials $E_{n} ( x ) $ are defined by means of the following generating series:

$$ E(x,z)=\frac{2}{e^{z}+1}e^{xz}=\sum_{n=0}^{\infty}E_{n} ( x ) \frac{z^{n}}{n!}\quad \bigl( \vert z \vert < \pi \bigr) . $$

In the case when $x=0$, we have $E_{n} ( 0 ) :=E_{n}$ that means associated Euler numbers given by

3.1 $$ E(z)=\frac{2}{e^{z}+1}=\sum_{n=0}^{\infty}E_{n} \frac {z^{n}}{n!},\quad \textit{cf. } \mbox{[9--11]}. $$

### Corollary 5

*The function* $E(z)$ *is a meromorphic function at everywhere*.

Let us now consider $E(z)$ over the following disk:

$$ D= \bigl\{ z\in \mathbb{C} \mid \vert z \vert \leq a \bigr\} . $$

The function $E(z)$ has no zeros. However, it has poles as follows:

$$ e^{z}+1=0\quad \Rightarrow\quad e^{z}=e^{2\pi i ( k-\frac{1}{2} ) } \quad \Rightarrow\quad z=2\pi i \biggl( k-\frac{1}{2} \biggr)\quad ( k\in \mathbb{Z} ) . $$

A number of poles over disk *D* are as follows:

- If $a=\pi$, the poles are $-\pi i$, *πi*: that is, $n ( a,E ) =2$ where $E:=E ( z ) $.
- If $a=3\pi$, the poles are $-3\pi i$, $-\pi i$, *πi*, $3\pi i$: that is, $n ( a,E ) =4$.

Then we have the following corollary.

### Corollary 6

*A number of poles of the function* $E ( z ) $ *over disk* $D= \{ z\in \mathbb{C}\mid \vert z \vert \leq a \}$:

$$ n ( a,E ) =2 \biggl[ \frac{a+\pi}{2\pi} \biggr] . $$

Now we give the following theorem.

### Theorem 7

*The function* $E ( z ) $ *holds for* $a=\pi$ *over disk* *D*

$$ N ( \pi,E ) =0. $$

### Proof

Since $E ( 0 ) \neq0,\infty$, it follows from Eq. (1.1) that

$$ N ( \pi,f ) = \int_{0}^{\pi}n ( t,E ) \frac {dt}{t}. $$

From Corollary 6, we have

$$ N ( \pi,E ) =2 \int_{0}^{\pi} \biggl[ \frac{t+\pi}{2\pi } \biggr] \frac{dt}{t}. $$

When $[ \frac{t+\pi}{2\pi} ] =0$ on $[ 0,\pi ) $, we deduce

$$ N ( \pi,E ) =0, $$

which completes the proof. □

Because of Theorem 7 and Definition 3, we have the following corollary.

### Corollary 7

$$ T ( \pi,E ) =m ( \pi,E ) . $$

### Theorem 8

*The mean value of the function* $E ( z ) $ *on* $[ 0,\pi ) $ *is that*

$$ m ( \pi,E ) =O ( 1 ) . $$

### Proof

As has been used in Theorem 5, we have

$$ \log^{+}\frac{1}{ \vert e^{z}-1 \vert }\leq\log^{+}\frac {1}{ \vert e^{\pi\cos\theta}-1 \vert } \quad \bigl( z=\pi e^{i\theta} \bigr) . $$

Then we write the mean value of the function $E ( z ) $ on $[ 0,\pi ) $

$$\begin{aligned} m ( \pi,E ) =&\frac{1}{\pi} \int_{0}^{\pi}\log ^{+}\frac{2}{ \vert e^{z}+1 \vert } \,d\theta \\ \leq&\frac{1}{\pi} \int_{0}^{\pi}\log^{+}\frac{1}{ \vert e^{\pi\cos \theta}-1 \vert } \,d\theta. \end{aligned}$$

Since

$$ e^{\pi\cos\theta}-1=\pi\cos\theta+\frac{ ( \pi\cos\theta ) ^{2}}{2!}+\cdots $$

with $\vert \cos\theta \vert \geq0$, we find

$$ \bigl\vert e^{\pi\cos\theta}-1 \bigr\vert \geq\pi \vert \cos\theta \vert \geq \vert \cos\theta \vert . $$

Then we have

$$\begin{aligned} m ( \pi,E ) \leq&\frac{1}{\pi} \int_{0}^{\pi}\log ^{+}\frac{1}{ \vert \cos\theta \vert } \,d\theta \\ =&\frac{1}{\pi} \int_{0}^{\pi}\log\frac{1}{ \vert \cos \theta \vert }\,d\theta. \end{aligned}$$

Here when the integral $\frac{1}{\pi}\int_{0}^{\pi}\log\frac{1}{ \vert \cos\theta \vert }\,d\theta$ is continuous, we get

$$ m ( \pi,E ) =O ( 1 ), $$

which is the desired result. □

### Theorem 9

*The Nevanlinna characteristic function of the function* $E ( z ) $ *is that*

$$ T ( \pi,E ) =O ( 1 ) . $$

### Proof

Since it follows from Definition 3, Theorem 7, and Theorem 8, we omit the proof. □

## Application

Let *f* be a meromorphic function in a domain $D\subset \mathbb{C}$. A function *f* is a *normal function* if there exists a positive number *K* such that

$$ f^{\sharp} ( \zeta ) \leq K $$

for any $\zeta\in D$, where

$$ f^{\sharp} ( \zeta ) =\frac{ \vert f' ( \zeta ) \vert }{1+ \vert f ( \zeta ) \vert ^{2}} $$

denotes the *spherical derivative* of *f*, *cf.* [7].

We now find spherical derivative of the function $\mathbf{B} ( z ) $ given by Eq. (2.1). Since

$$ \mathbf{B}' ( z ) =-\frac{e^{z}}{ ( e^{z}-1 ) ^{2}}, $$

we have

$$ \mathbf{B}^{\sharp} ( z ) =\frac{ \vert \frac {e^{z}}{ ( e^{z}-1 ) ^{2}} \vert }{1+ \vert \frac{1}{ ( e^{z}-1 ) ^{2}} \vert }=\frac{\frac{ \vert e^{z} \vert }{ \vert e^{z}-1 \vert ^{2}}}{1+\frac{1}{ \vert e^{z}-1 \vert ^{2}}}= \frac{ \vert e^{z} \vert }{1+ \vert e^{z}-1 \vert ^{2}}. $$

Here we consider $z=re^{i\theta}$ with $-\frac{\pi}{2}\leq\theta \leq \frac{\pi}{2}$ and $r>1$. Then it becomes

$$ \mathbf{B}^{\sharp} ( z ) =\frac{ \vert e^{z} \vert }{1+ \vert e^{z}-1 \vert ^{2}}=\frac{e^{r\cos\theta }}{1+ \vert e^{re^{i\theta}}-1 \vert ^{2}}. $$

By the triangle inequality $\vert \vert z_{1} \vert - \vert z_{2} \vert \vert \leq \vert z_{1}-z_{2} \vert $, we have

$$ \mathbf{B}^{\sharp} ( z ) \leq\frac{e^{r\cos\theta }}{1+\cos ^{2}\theta}. $$

It is easy to see that

$$ e^{r\cos\theta}-1=r\cos\theta+\frac{ ( r\cos\theta ) ^{2}}{2!}+\cdots\geq r\cos \theta\geq\cos\theta. $$

We derive

$$ \mathbf{B}^{\sharp} ( z ) \leq\frac{e^{r\cos\theta } ( e^{r\cos\theta}+1 ) }{1+\cos^{2}\theta}. $$

Because of the inequality $\frac{1}{2}\leq\frac{1}{1+\cos^{2}\theta }\leq 1 $, we reach the following inequality:

$$ \mathbf{B}^{\sharp} ( z ) \leq e^{r} \bigl( e^{r}+1 \bigr) . $$

Since *r* is a real number greater than 1, we can write $K:=e^{r} ( e^{r}+1 ) $ as follows

$$ \mathbf{B}^{\sharp} ( z ) \leq K. $$

Thus we get the following theorem.

### Theorem 10

*The function* $\mathbf{B} ( z ) =\frac{1}{z}+\sum_{n=0}^{\infty}\frac{B_{n+1}}{n+1}\frac{z^{n}}{n!}$ *is a normal function*.

## Conclusion and observation

Although Nevanlinna theory was studied intensively in complex analysis by many mathematicians, it was not considered to apply the tools of Nevanlinna theory to generating functions of special numbers. This work was first done in this issue. We dealt mainly with the generating functions on Nevanlinna theory. It seemed interesting with the following properties:

$$ T ( 2\pi,\mathbf{B} ) =\log ( 2\pi ) +O ( 1 )\quad \text{and}\quad T ( \pi,E ) =O ( 1 ) . $$

In fact, the identities of special numbers have been studied in great detail. From some of relations, it is possible to obtain further properties on Nevanlinna theory by making use of some of the identities. For example,

5.1 $$ \sum_{n=0}^{\infty}G_{n} \frac{z^{n}}{n!}=\frac{2z}{e^{z}+1} $$

is known as a generating function of Genocchi numbers, denoted by $G_{n}$. Comparing Eq. (3.1) with Eq. (5.1), one can easily derive

$$ E_{n}=\frac{G_{n+1}}{n+1},\quad \textit{cf. } \mbox{[9--11]}. $$

By using this relation, one may derive easily the Nevanlinna characteristic function of the generating function of Genocchi numbers the same as the Nevanlinna characteristic function of the generating function of associated Euler numbers.
